# Psychometric validation of the nursing care behavior questionnaire during emerging disease epidemics: A theory of planned behavior approach

**DOI:** 10.1016/j.heliyon.2024.e25900

**Published:** 2024-02-09

**Authors:** Afsaneh Raiesifar, Reyhaneh Maleki, Nasibeh Sharifi, Fatemeh Darabi, Sayyadi Hojjat

**Affiliations:** a Department of Nursing, School of Nursing & Midwifery, Ilam University of Medical Sciences, Ilam, Iran; b Department of Nursing, School of Nursing & Midwifery, Ilam University of Medical Sciences, Ilam, Iran; c Department of Midwifery, School of Nursing & Midwifery, Ilam University of Medical Sciences, Ilam, Iran; d Department of Publice Health, Asadabad School of Medical Sciences, Asadabad Iran; e Non-Communicable Diseases Research Center, Ilam University of Medical Sciences, Ilam, Iran

**Keywords:** Psychometrics, Nurses, Emerging diseases, Item-response theory

## Abstract

**Background and objective:**

The Covid-19 pandemic could compromise the quality of care by impacting nurses' intention to provide care. Hence, investigating nurses' behavioral intentions when caring for patients during epidemics is essential. This research aims to assess the psychometrics of the “Nursing Care Behavior in Epidemics of Emerging Diseases” questionnaire, based on the constructs of the Theory of Planned Behavior.

**Methods:**

This cross-sectional study involved 311 nurses working in Covid-19 wards of selected hospitals in Ilam City, all of whom were enrolled through a census in 2021. The questionnaire underwent initial translation from English to Persian, followed by evaluations of its face, content, and construct validities. The nursing caring behavior questionnaire, comprising 46 items, was designed to assess behavioral beliefs, normative beliefs, control beliefs, behavioral attitudes, subjective norms, perceived behavioral control, and nurses' intentions to care for Covid-19 patients. Data were analyzed for face, content, and construct validity using the Theory of Planned Behavior and Pearson correlation. Reliability was determined by calculating the Cronbach's alpha coefficient.

**Results:**

The intention to care dimension demonstrated an inverse correlation with the behavioral attitude dimension (p < 0.001). The most robust correlations were observed between the following paired dimensions: behavioral attitude and perceived power (p < 0.001), subjective norms and intention to care (p < 0.001), perceived behavioral control and subjective norms (p < 0.001), care intention and behavioral beliefs (p < 0.001), behavioral beliefs and behavioral outcome evaluation (p < 0.001), and normative beliefs with motivation to comply (p = 0.001). The Cronbach's alpha coefficient of the instrument exceeded 0.75.

**Conclusion:**

According to the Theory of Planned Behavior, the nursing care behavior questionnaire proved to be a valid and reliable tool for evaluating nurses' care behaviors amidst emerging disease epidemics.

## 1 Introduction

A novel form of coronavirus disease, known as Covid-19, was initially identified in China in December 2019. The rapid spread of this pandemic to various countries placed substantial physical and mental burdens on hospitalized patients, their families, healthcare personnel, and healthcare systems [1,2]. Consequently, a global mobilization of public health initiatives ensued.

Caring is intrinsically imperative, encompassing a spectrum of actions and behaviors delivered by nurses to patients across physical, social, psychological, emotional, and spiritual dimensions [3,4]. During epidemics, providing care to patients places considerable stress on nurses, often triggering negative psychological responses including depression, anxiety, and irritability. This psychological toll is particularly pronounced among nurses tasked with caring for Covid-19 patients [5]. Occupational pressure, stress, and anxiety may drive certain nurses to leave their positions, creating adverse consequences for the healthcare system as a whole. Pressures stemming from emerging infectious diseases prompt nurses in hospital settings to experience fear and apprehension towards their care responsibilities, sometimes causing them to neglect this crucial role [6].

The Covid-19 pandemic jeopardizes the quality of care by influencing nurses' intent to care for Covid-19 patients [7]. These sudden and disruptive epidemics can impact nursing care and nurses' behavioral intentions during such crises, divulging nurses' behavioral intention to care for patients can yield valuable insights for enhancing patient care during future epidemics [8,9]. caring behaviors, in turn, consist of data, abilities, attitudes, and essential values for the accomplishment of professional actions. Therefore, investigating nurses' attention of caring behaviors allows nursing staffs and administrators to receptively plan for enjoyable all the necessities of clients [10,11]. Before any making plans, the behavior needs to be as it should be perceived. behavior may be properly elucidated the usage of the to be had theories and models.

An organized and specific health framework is instrumental in thoroughly examining the issue and conducting a comprehensive conceptual analysis. Established models and theories play a vital role in assessing nursing care behaviors [12,13]. Meta-analysis research of the theory of planned behavior (TPB) have discovered that the TPB's construct properly predicts intention after which behavior [13]. The planned behavior theory, developed by Ajzen, is rooted in the reasoned action theory and forecasts the occurrence of a specific behavior based on an individual's intent to engage in that behavior. This theory posits that behavioral intention is determined by three factors: attitude toward the behavior, subjective norms, and perceived behavioral control [11,12].

Numerous studies have explored the applicability of the planned behavior theory in elucidating nurses' intentions to care for Covid-19 patients [6,14]. These investigations employed a questionnaire based on the theory of planned behavior, developed by Lee et al. (2020) [5] and adapted it for caring for Covid-19 patients. Similarly, a psychometrically validated tool designed for SARS patients by Yoo et al. in South Korea in 2005 [15] was also employed. Both studies affirmed the internal consistency and favorable reliability of the scale. Developed by Ajzen in 1988 as an extension of the reasoned action theory to offer a standard questionnaire based on the theory of planned behavior to be employed to predict the behaviors of a specific population worldwide [16]. There is a need to develop a highly reliable and valid tool to predict the behavior of nurses around the world, especially their behaviors when caring for Covid-19 patients. The development of such a questionnaire based on the theory of planned behavior can pave the path for future interventions and research efforts. Therefore, the present study aimed to evaluate the psychometrics properties of the nurses’ caring behavior questionnaire during the epidemics of emerging diseases based on the theory of planning behavior among Iranian nurses.

## 2 Methods

Study Design and Participants: This methodological study aimed to psychometrically validate the nursing care behavior questionnaire concerning the care of Covid-19 patients during the pandemic, utilizing the theory of planned behavior during 2020. The study enrolled 311 nurses employed in teaching hospitals affiliated with the Ilam University of Medical Sciences. Participants were selected through a census approach and based on including criteria (having bachelor degree in nursing or above, work experiences in covid wards ≥4 months and intent to participate in the study). If the questionnaires were not completed or incomplete, Participants were excluded from the study, Due to the fact that the questionnaire was completed online and the answer to each question was mandatory in the questionnaire settings, and it was possible to register only at the end of the complete completion of the questionnaire; Therefore, all registered questionnaires were complete and no questionnaires were excluded.

### 2.1 Instruments

A. Demographic Questionnaire: This questionnaire encompassed inquiries about age, gender, educational level, marital status, organizational position, hospital of employment, specific ward within the hospital, work experience, employment type, experience in providing care during emerging infectious disease epidemics, and history of training related to personal protective equipment and emerging infectious diseases.
B. Nurses' Caring Behavior Scale: Developed by Lee et al., in 2019, this scale comprises 46 items, each rated on a seven-point Likert scale, ranging from complete disagreement to complete agreement. The dimensions of the tool encompass behavioral beliefs (18 items), normative beliefs (8 items), control beliefs (10 items), behavioral attitudes (3 items), subjective norms (2 items), perceived behavior control (2 items), and intention to care for Covid-19 patients (2 items). The scale's final mean score was calculated. A score of 1 indicated total agreement, while a score of 7 indicated complete disagreement. The psychometric validation of the scale followed the International Quality of Life Assessment (IQOLA) translation method [17], involving the following steps.

**Validation Process:** This validation process encompassed the standard translation of the questionnaire from English to Persian, followed by assessments of the tool's face validity, content validity, and construct validity.

### 2.2 Translation process

Before creating the Persian version of the questionnaire, we obtained the developer's (Lee) permission. Lee designed and introduced the tool in 2019 to assess factors influencing nurses' willingness to care for patients with emerging diseases, based on the theory of planned behavior. Subsequently, the questionnaire underwent separate translation into Persian by two proficient experts in English and Persian. Through the comparison of these two versions, a final draft of the Persian questionnaire was prepared (i.e., the forward stage). Subsequently, this Persian version underwent reverse translation from Persian to English by two English-fluent individuals who were unaware of the original questionnaire's content. The study supervisor then compared the content of this translated version to the original questionnaire (i.e., the backward step). After obtaining approval from the main author, the final translated questionnaire version was compiled.

### 2.3 Content and face validity

To assess face validity, the Persian version of the questionnaire was presented to a group of nurses and field specialists to solicit their opinions on the questionnaire's appearance.

For evaluating content validity, the Persian questionnaire was shared with ten experts, including nursing professors, specialists in infectious and pulmonary diseases, and behavioral science experts. Their input was sought to gauge the questionnaire's content. During this phase, both the content validity index and content validity ratio were computed. Expert feedback from this phase led to the addition of 22 questions to the scale. These questions comprised 4, 1, 3, 1, 5, 5, and 3 questions across self-reported behavioral beliefs, the subjective norms, perceived behavioral control, nursing intention, power over controlling factors, motivation to comply, and behavioral outcome evaluation dimensions, respectively.

Additionally, following the expert team's advice, an extra section was included in the questionnaire for behavioral assessment, encompassing the following questions:

1. Have you ever provided care to a Covid-19 patient?
2. If you answered yes to the previous question, how long was your caregiving duration?
3. Do you intend to care for Covid-19 patients within the next six months? [ ] Yes [ ] No
4. Do you intend to care for Covid-19 patients within the next twelve months? [ ] Yes [ ] No

**Fig. 1:**
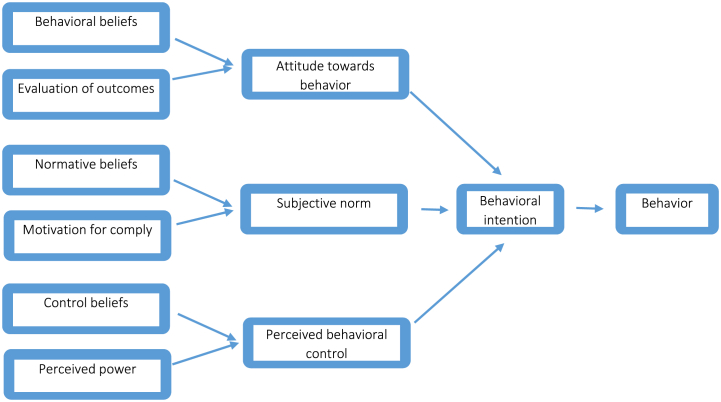
Theory of planned behavior.

In the initial TPB-based questionnaire, after the demographic questions, there were 4 basic questions about self-reported behavioral beliefs (4 items) during the covid-19 pandemic; followed by 11 questions (questions 1 to 11) about behavioral beliefs about caring for patients with covid 19, 11 questions (questions 12 to 22) about evaluation of outcomes, 5 questions (questions 23 to 27) about normative beliefs (abstract norms) about caring for patients with COVID19, 5 questions (questions 28 to 32) about Motivation of comply, 8 questions (questions 33 to 40) about control beliefs (perceived control) about caring for patients with COVID19, 9 questions (Questions 41 to 49) about perceived power, 3 questions (Questions 50 to 52) Attitude towards the care behavior of patients with COVID19, 3 questions (Questions 53 to 55) Mental norms about the care of patients with COVID19, 5 questions (Question 56 to 60) direct measurement of perceived behavioral control regarding the care of patients with COVID19 and 4 questions (questions 61 to 64) were the intention of nurses to care for patients with COVID19. Conceptual model of theory of planed behavior shows in Fig. 1.

After the item response theory (IRT) analysis, six questions were removed from the initial questionnaire and 58 questions were finalized, which are fully explained in the findings section, Table No. 3.

### 2.4 Construct validity

**Evaluation of Construct Validity:** In this study, we utilized the item-response theory to assess the construct validity of the tool. To achieve this, ethical clearance (ethics code: IR.MEDILAM.REC.1399.257) was obtained from the Research Ethics Committee of Ilam University of Medical Sciences. A roster comprising the names of nurses employed in three teaching university-affiliated hospitals within Ilam city was compiled. Subsequently, the study's objectives were communicated to the nurses, informed consent was secured, and the online Persian version of the nursing care behavior questionnaire (comprising 64 items) was administered to gather the required data. A total of 311 nurses working in teaching university-affiliated hospitals in Ilam city, possessing over four months of work experience and holding bachelor's degrees or higher, were willingly included in the study. These participants were selected through a census approach, while those who submitted incomplete questionnaires were excluded from the final analysis.

### 2.5 Reliability assessment (internal consistency and stability)

We employed internal consistency using Cronbach's alpha method to gauge the questionnaire's reliability. To this end, the questionnaire was distributed among 30 eligible nurses who participated in the study and forementioned coefficient was calculated. These nurses were selected by simple random selection and have same demographic criteria with nurses for the main study. These 30 nurses were excluded the main study.

### 2.6 Ethical considerations

Necessary permissions for conducting this study were obtained from the authorities at Ilam University of Medical Sciences, as well as from officials at Shahid Mostafa Khomeini, Imam Khomeini, and Ayatollah Taleghani hospitals. Initially, the nurses were briefed about the research's objectives, and those who agreed to participate were asked to sign a written informed consent form. The nurses who met the inclusion criteria were then provided with the questionnaires for completion. This article presents the outcomes of a research project endorsed by the Research Council of Ilam University of Medical Sciences and the institutional ethics committee under the reference code IR.MEDILAM.REC.1399.257.

### 2.7 Statistical analysis

Data analysis was carried out using STATA 11 software (Stata Corp., LLC College Station, Texas, USA). Descriptive statistics, including measures of centrality and dispersion, were used to characterize quantitative variables. Given that the items exhibited a non-normal distribution and are in Likert scale, construct validity was evaluated using the item response theory (IRT) analysis with the graded response model. A significance threshold of 5% was applied for all tests.

## 3 Results

The participants' mean age was 27.07 ± 4.85 years, with a standard deviation. Among the subjects, 51.1% were females, 62.4% held bachelor's degrees, and 32.2% had experience in providing nursing care to patients with infectious emerging diseases (Table 1).

**Table 1:** General characteristics of participants.

Variables	Characteristics	Frequency (percentage)
**Age**	<30	(78.8)
	>30	(21.2)
**Sex**	Male	(48.9)
	Female	(51.1)
**Education**	Bachelor	(62.4)
	Master	(37.6)
**Previous care experience on emerging infectious diseases**	Yes	(30.2)
	No	(69.8)
**Work experience**	<59 Month	(37.9)
	>59 Month	(62.1)

As indicated in Table 2, the dimensions exerting the most and least influence on nurses' caring behaviors were behavioral attitude (encompassing behavioral beliefs and behavioral outcome assessment) with a mean score of 37.49 ± 6.00, and subjective norms (including normative beliefs and motivation to comply) with a mean score of 9.84 ± 1.69.

**Table 2:** The scores related to the constructs of the nursing care behavior questionnaire based on the theory of planned behavior.

Dimensions	Mean ± SD
Attitude toward behavior (questions 45 to 47)	15.84 ± 4.02
Behavioral beliefs (questions 1 to 10)	37.49 ± 6
Evaluation of behavioral outcome (questions 11 to 21)	32.58 ± 6.82
Total behavioral attitude	70.07 ± 11.74
Subjective norms)questions 48 to 50)	9.84 ± 1.69
Normative beliefs (questions 22 to 25)	13.12 ± 4.29
Motivation to comply (questions 25 to 28)	13.35 ± 2.57
Total subjective norms	26.47 ± 5.39
Perceived behavioral control)questions 51 to 55)	13.81 ± 3.65
Control beliefs (questions 29 to 35)	26.67 ± 6.52
Perceived power (questions 36 to 44)	31.07 ± 6.71
Total behavioral control	55.72 ± 10.74
Total intention to care (questions 56 to 58)	39.51 ± 6.43

In terms of the questionnaire's content validity, each question's Content Validity Ratio (CVR) and Content Validity Index (CVI) were calculated. The calculated CVR values ranged from 0.9 to 0.99, and the CVI values spanned from 0.6 to 0.98. Among the questions, the highest CVR score was associated with Question No. 2 (0.99), while Question No. 14 received the lowest CVR score (0.77). Similarly, in terms of CVI, Question No. 10 obtained the highest score (0.99), while Question No. 4 received the lowest CVI score (0.86).

Table 3 presents the outcomes of assessing the statistical significance concerning the relationship between the queries and their respective dimensions using IRT analysis. The standard errors and *P*-values are displayed at the 5% significance level for all tests. Within the dimension of behavioral beliefs (encompassing questions 1 to 11), Question No. 9 was excluded. In the span of questions 12 to 22, no items were removed. For questions 23 to 27, which are associated with the normative beliefs dimension, Question No. 25 was eliminated from the questionnaire. In relation to questions 28 to 32, pertaining to the motivation to comply dimension, Questions No. 31 and 32 were taken out. Within the scope of questions 33 to 40, associated with the control beliefs dimension, one item (Question No. 39) was omitted. Lastly, from the questions pertaining to nursing intention (items 61 to 64), Question No. 61 was removed due to its P value exceeding 0.05. Ultimately, a total of 58 questions were retained within the questionnaire.

**Table 3:** The significance levels and standard errors related to the queries embedded in the nursing behavior questionnaire using IRT analysis with the graded response model.

Questions	Coefficient	Std.err	*P*-Value
Q1	o.39	0.12	0.002
Q2	0.33	0.12	0.007
Q3	−1.65	0.23	<0.001
Q4	−0.46	0.12	<0.001
Q5	1.33	0.17	<0.001
Q6	2.81	0.48	<0.001
Q7	0.21	0.12	0.076
Q8	−0.57	0.12	<0.001
**Q9**	**0.09**	**0.12**	**0.447**
Q10	−0.20	0.11	<0.001
Q11	1.78	0.22	<0.001
Q12	1.39	0.16	<0.001
Q13	0.20	0.11	0.075
Q14	−1.14	0.14	<0.001
Q15	−0.27	0.11	0.017
Q16	3.08	0.30	<0.001
Q17	1.21	0.14	<0.001
Q18	3.17	0.31	<0.001
Q19	5.51	0.92	<0.001
Q20	0.87	0.13	<0.001
Q21	0.34	0.10	0.002
Q22	0.91	0.13	<0.001
Q23	0.20	0.13	0.029
Q24	0.74	0.14	<0.001
**Q25**	**0.02**	**0.10**	**0.777**
Q26	9.18	2.50	<0.001
Q27	1.67	0.20	<0.001
Q28	2.09	0.39	<0.001
Q29	1.81	0.32	<0.001
Q30	1.73	0.28	<0.001
**Q31**	**0.13**	**0.15**	**0.367**
**Q32**	**−0.05**	**0.13**	**0.367**
Q33	0.94	0.14	<0.001
Q34	3.04	0.49	<0.001
Q35	0.88	0.14	<0.001
Q36	2.13	0.25	<0.001
Q37	2.48	0.36	<0.001
Q38	0.78	0.13	<0.001
**Q39**	**−0.22**	**0.14**	**0.118**
Q40	1.73	0.23	<0.001
Q41	0.47	0.12	<0.001
Q42	−1.19	0.14	<0.001
Q43	4.14	0.44	<0.001
Q44	2.70	0.27	<0.001
Q45	4.04	0.45	<0.001
Q46	−0.81	0.13	<0.001
Q47	1.71	0.17	<0.001
Q48	3.22	0.33	<0.001
Q49	1.13	0.14	<0.001
Q50	1.31	0.10	0.002
Q51	2.20	0.18	<0.001
Q52	1.80	0.10	<0.001
Q53	0.40	0.13	0.002
Q54	−5.33	1.68	0.002
Q55	1.05	0.15	<0.001
Q56	2.11	0.22	<0.001
Q57	5.16	1.59	0.001
Q58	4.39	1.18	<0.001
Q59	0.73	0.14	<0.001
Q60	−0.54	0.13	<0.001
**Q61**	**0.06**	**0.11**	**0.546**
Q62	−0.64	0.14	<0.001
Q63	5.88	2.39	0.001
Q64	1.24	0.18	<0.001

The bold text means that the item was excluded from the final questionnaire due to its P value exceeding 0.05.

**Fig. 2:**
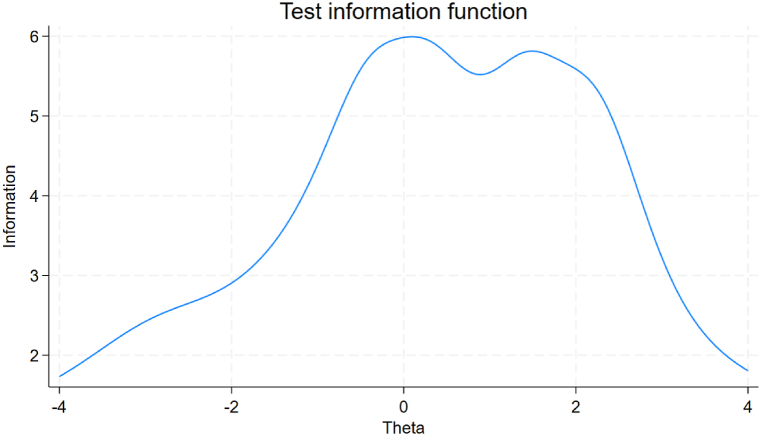
Test information function.

Further, the below figure of Test information function (TIF) suggests that the scale consisting of the 10 items (items 1–11 except 9) under consideration could be used as an instrument evaluating with highest precision after excluding the nineth item (figure-2).

In terms of the interrelationships within the nursing behavior questionnaire components, based on the theory of planned behavior, the most substantial correlation coefficients were found between the following dimensions: behavioral attitude and perceived power (r = 0.722, p < 0.001), subjective norms and care intention (r = 0.288, p < 0.001), care intention and attitude towards behavior (r = −0.426, p < 0.001), care intention and behavioral beliefs (r = 0.404, p < 0.001), behavioral beliefs and behavioral outcome assessment (r = 0.676, p < 0.001), normative beliefs and motivation to comply (r = 0.186, p = 0.001), control beliefs and behavioral beliefs (r = 0.539, p < 0.001), behavior evaluation and behavioral beliefs (r = 0.676, p < 0.001), motivation to comply and normative beliefs (r = 0.186, p = 0.001), and finally, perceived power and attitude towards behavior (r = 0.722, p < 0.001) (Table 4).

**Table 4:** Correlation coefficients between components of the theory of planned behavior questionnaire.

Dimension		Attitude toward behavior	Subjective norm	Perceived behavioral control	Intention to care	Behavioral belief	Normative belief	Control belief	Evaluation of behavioral outcome	Motivation of Comply	Perceived power
**Attitude toward behavior** (questions 45 to 47)	Pearson (p-value)	1	0.004 (0.949)	0.182 (0.001)	−0.426 (0.000)	0.039 (0.492)	−0.165 (0.004)	0.321 0.000	0.394 (0.000)	−0.146 (0.010)	0.722 (0.000)
**Subjective norm** (questions 48 to 50)			1	0.288 (0.000)	0.168 (0.003)	0.077 (0.177)	0.096 (0.089)	0.069 (0.228)	0.194 (0.001)	−0.075 (0.188)	0.065 (0.253)
**Perceived behavioral control** (questions 51 to 55)				1	0.194 (0.001)	−0.009 (0.876)	−0.074 (0.193)	0.177 (0.002)	0.043 (0.447)	−0.110 (0.052)	0.183 (0.0010
**Intention to care** (questions 56 to 58)					1	0.404 (0.000)	0.112 (0.049)	0.284 (0.000)	0.089 (0.116)	0.091 (0.108)	−0.203 (0.000)
**Behavioral belief** (questions 1 to 10)						1	−0.059 (0.300)	0.539 0.000	0.676 (0.000)	−0.45 (0.424)	0.116 (0.041)
**Normative belief** (questions 22 to 25)							1	−0.025 (0.666)	−0.175 (0.002)	0.186 (0.001)	−0.001 (0.990)
**Control belief** (questions 29 to 35)								1	0.433 (0.000)	−0.112 (0.048)	0.318 (0.000)
**Evaluation of behavioral outcome** (questions 11 to 21)									1	−0.134 (0.118)	0.150 (0.008)
**Motivation of Comply** (questions 25 to 28)										1	−0.117 (0.039)
**Perceived** **Power** (questions 36 to 44)											1

Cronbach's alpha coefficients and Intraclass Correlation Coefficients (ICC) are listed in Table 5 for each dimension of the theory of planned behavior questionnaire. The highest and lowest Cronbach's alpha coefficients were associated with the dimensions of attitude towards behavior (0.79) and normative beliefs (0.35), respectively.

**Table 5:** Cronbach's alpha coefficients obtained for various dimensions of the theory of planned behavior questionnaire.

Dimensions	Cronbach's alpha coefficient
Attitude toward behavior	0.79
Subjective norm	0.49
Perceived behavioral control	0.63
Intention to care	0.40
Behavioral belief	0.64
Normative belief	0.35
Control belief	0.68
Evaluation of behavioral outcome	0.78
Motivation of comply	0.40
Perceived power	0.85
Total	0.94

## 4 Discussion

Caring, as a universal imperative, resonates with humanity's inherently social nature and underscores the bedrock of human relationships. It holds a pivotal place in nursing, providing a moral compass that upholds and elevates human values. Nursing care behavior comprises a diverse array of constructs [18], necessitating the utilization of valid tools rooted in educational models for effective determination. The present study aimed to evaluate the psychometric properties of the Persian version of the nursing care behavior questionnaire, tailored to emerging diseases through the lens of the theory of planned behavior, in order to contextualize it within Iran's culture.

In this research, the studied questionnaire unveiled a multifaceted construct, which was suitably modified from its original version, following the permission of the developer, to align with the Iranian context. Following the endorsement of the Persian translated questionnaire, its validity and reliability were meticulously examined, indicating that this scale indeed emerges as a reliable instrument for gauging nurses' care behaviors during epidemics. The questionnaire was affirmed to have content, face, and construct validities. Questions' content validity index ranged from 0.9 to 1, with two questions being excluded due to a content validity index below 0.6. A supplementary 4-item section was introduced to the scale for behavior evaluation, based on input from the validation process. Subsequently, the item-response theory was employed to evaluate the questionnaire's construct validity. Questions No. 9, 25, 39, and 61 were excluded due to P values surpassing 0.05, affecting the dimensions of behavioral beliefs (questions 1 to 11), normative beliefs (questions 23 to 27), control beliefs (questions 33 to 40), and nursing intention (questions 61 to 64), respectively. Additionally, questions 31 and 32 were omitted from the motivation to comply dimension. Consequently, a modified 58-item questionnaire, harmonized with Iran's context, was established. The tool's reliability was assessed through Cronbach's alpha coefficient, yielding a robust value of 0.94 for the entire instrument, underscoring its desirable internal consistency. The items' variance mirrored the theoretical construct of the main questionnaire, with insignificant residual or measurement errors. These errors, inherent to research tools to a certain extent, are unrelated to concealed contextual constructs [[19], [20], [21]]. The limited residual error reaffirmed the reliability and validity of the studied tool, offering consistent outcomes across consecutive applications. Furthermore, the substantial correlation coefficients within each dimension underscored their interconnectivity.

Yoo et al. (2005) conducted a study on the validity and reliability of a tool predicting nurses' intent to care for SARS patients, confirming its high validity and reliability. Similarly, in the present study, factor analysis corroborated six factors, validating the questionnaire as an apt instrument for nursing managers and researchers to assess clinical nurses' prominent beliefs regarding care provision to patients with infectious diseases. It aids in devising effective intervention strategies and evaluating their efficacy [15].

Lee et al.'s study highlighted the satisfactory validity and utility of the mentioned questionnaire in measuring nurses' care behaviors toward patients with infectious diseases, exhibiting strong correlations among its dimensions [5].

This study's evaluation of a behavioral assessment tool based on the theory of planned behavior showcases its potential in predicting nurses' intent to care for patients afflicted by emerging infectious diseases. Another study corroborated the theory's efficacy in predicting nurses' intent toward caring for such patients [5]. Consequently, the theory of planned behavior serves as a normative framework to identify expected behaviors and uphold professional nursing and moral values across all nurses [5,22].

According to the study's findings, 29.4% of the enrolled nurses exhibited negative caring behavior, while 70.6% displayed positive caring behavior. Various factors, including age, marital status, organizational position, training history on personal protective equipment, type of employment, and work experience, were associated with nurses' intent to care for Covid-19 patients.

In a study by Cheng et al. (2021) on factors influencing nurses' behavioral intent toward caring for Covid-19 patients in China, behavioral attitudes, subjective norms, perceived behavioral control, and work experience significantly influenced nurses' intent [7]. Similarly, Aljohani et al. (2021) in Saudi Arabia reported that nurses' high intent to care for Covid-19 patients correlated with educational level, history of ongoing training on emerging infectious diseases, and job position (e.g., being a supervisor) [23]. Pesridis et al. (2021) in Greece revealed higher scores in behavioral intention, subjective norms, and perceived behavioral control among nurses caring for Covid-19 patients. Healthcare workers with prior experience in caring for Covid-19 patients exhibited a higher intent to continue their role, echoing the present study's results [24].

In summary, this study's comprehensive assessment of the nursing care behavior questionnaire within the theory of planned behavior demonstrates its applicability in predicting nurses' intent to care for patients with emerging infectious diseases. This framework proves valuable in identifying anticipated behaviors and upholding nursing values, fostering the crucial endeavor of caring for patients during epidemics.

### 4.1 Strength and limitation of the study

Due to the fact that pandemics similar to covid-19 may occur in the future and due to the importance of informing managers and policymakers about nurses' care behaviors as the front line of dealing with these pandemics, this tool as a reliable and valid tool for assessing the situation and taking urgent action can be used in other similar pandemics. The impossibility of random sampling due to the limited statistical population in Ilam city is one of the most important limitations of this study. Also, the results of the study were selected from the cities of Ilam, this sample does not represent variation of all the country population.

## 5 Conclusion

The outcomes of this current study, which aimed to adapt the Persian version of the nursing care behavior questionnaire for emerging diseases using the theory of planned behavior, underscored its robust internal consistency and satisfactory validity and reliability. Notably, this study marked the pioneering endeavor in Iran to comprehensively examine the face, content, and construct validities of this tool. Furthermore, the results from the confirmatory factor analysis provided substantive validation for this questionnaire's utility, thus enabling researchers to readily incorporate the Persian version of this established questionnaire in their investigative pursuits.

## Funding

This article emanated from an approved master's thesis conducted at Ilam University of Medical Sciences (Grant NO 993009/142). The authors unequivocally affirm that they did not receive any funding from any external organization.

## Data availability statement

The study data will be provided to the researchers upon their request to correspond author.

## CRediT authorship contribution statement

**Afsaneh Raiesifar:** Writing – original draft, Validation, Methodology, Funding acquisition, Data curation, Conceptualization. **Reyhaneh Maleki:** Writing – original draft, Methodology, Funding acquisition. **Nasibeh Sharifi:** Supervision, Methodology, Funding acquisition, Conceptualization, Data curation, Validation. **Fatemeh Darabi:** Resources, Investigation, Conceptualization. **Sayyadi Hojjat:** Methodology, Investigation, Data curation, Conceptualization, Sofware, Formal analysis, Validation.

## Declaration of competing interest

The authors declare that they have no known competing financial interests or personal relationships that could have appeared to influence the work reported in this paper.

## References

[1] Byon H.D. (2022). Nurses' experience with type II workplace violence and underreporting during the COVID-19 pandemic.

[2] Chow E.J., Uyeki T.M., Chu Helen Y. (2023). The effects of the COVID-19 pandemic on community respiratory virus activity. Nature Rev. Microbiol..

[3] Babapour A.-R., Gahassab-Mozaffari N., Fathnezhad-Kazemi A.J.B.n. (2022). Nurses’ job stress and its impact on quality of life and caring behaviors: A cross-sectional study. BMC Nursing.

[4] Duffy J.R. (2022).

[5] Lee J., Kang S.J.J.N., Sciences H. (2020). Factors influencing nurses' intention to care for patients with emerging infectious diseases: Application of the theory of planned behavior. Nurs. Health Sci..

[6] Khalid A.A. (2021). Understanding factors contributing to nurses' intention to care for COVID-19 patients using the theory of planned behavior. Sudan J. Med. Sci. (SJMS).

[7] Cheng J. (2021). Factors influencing nurses' behavioral intention toward caring for COVID-19 patients on mechanical ventilation: a cross-sectional study. PLoS One.

[8] Lovrić R. (2020). Studying during the COVID-19 pandemic: A qualitative inductive content analysis of nursing students’ perceptions and experiences. Educat. Sci..

[9] Mo Y. (2020). Work stress among Chinese nurses to support Wuhan in fighting against COVID‐19 epidemic. J. Nurs. Manag..

[10] Balejani A A.N., Hoseinloo A. (2012). A survey on nurses' perception of the importance of caring behaviors and factors affecting its provision. Evidance Based Care J..

[11] Kim C.J., H Y., Yoo M.S., Kwon B.E., Hwang K.J. (2006). Attitude, beliefs, and intentions to care for SARS patients among Korean clinical nurses: an application of theory of planned behavior. J. Korean Acad. Nurs..

[12] Taleghani F A.N. (2015). Nursing basic concepts of islam thought: a conceptual model. Iran. J. Nat. Resour..

[13] Moradian Azin S R.N., Khorsandi M. (2016). The assessment of the theory of planned behavior on blood donation behaviors of the staff. Sci. J. Iran Blood Transfus. Organ.

[14] Al Maskari T.S. (2022). Using the Theory of Planned Behaviour to assess nursing and allied health students' knowledge and intention to care for patients with COVID-19. J. Clin. Nurs..

[15] Yoo H.R. (2005). [Validity and reliability of an instrument for predictive nursing intention for SARS patient care]. Taehan Kanho Hakhoe Chi.

[16] K M. (2012). Psychometric properties of a theory of planned behavior questionnaire for tobacco use in male adolescents. Quarterly J. Sabzevar Univ. Med. Sci..

[17] Gandek B., Ware J.E. (1998). Methods for validating and norming translations of health status questionnaires: the IQOLA Project approach. International Quality of Life Assessment. J. Clin. Epidemiol..

[18] Leyva E.W.A. (2015). Global perspectives of caring: an integrative review. Int. J. Human Caring.

[19] Patelarou A.E. (2020). Development and validation of a questionnaire to measure knowledge of and attitude toward COVID-19 among nursing students in Greece. Nursing Reports.

[20] Shohani M., Tavan H. (2018). The validity and reliability of the constructs of pain management-measuring tool for incurable patients. Iranian Red Crescent Med. J..

[21] Tavan H. (2015). Factor analysis of spiritual health on the islam viewpoint. Iranian J. Public Health.

[22] FerenczKaddari M., Shifman A., Koslowsky M. (2016). Modeling psychologists' ethical intention: application of an expanded theory of planned behavior. Psychol. Rep..

[23] Aljohani K.A. (2021). Understanding factors contributing to nurses’ intention to care for COVID-19 patients using the theory of planned behavior. Sudan J. Med. Sci..

[24] Pesiridis T. (2021). Providing care to patients with COVID-19 in a reference hospital: health care staff intentional behavior and factors that affect it. AIMS Public Health.

